# Trends in sales of sugar-sweetened beverages and associated type 2 diabetes burden in nine African countries: an ecological time-series analysis

**DOI:** 10.1080/16549716.2025.2568283

**Published:** 2025-10-09

**Authors:** Caroline H. Karugu, Gershim Asiki, Senzo Mthembu, Samuel Iddi, Peter M. Kaberia, Shukri F. Mohamed, Richard E. Sanya, Sylvia Kiwuwa-Muyingo, Stefanie Vandevijvere, Charles Agyemang

**Affiliations:** a Chronic Diseases Management Unit, African Population Health Research Center, Nairobi, Kenya; b Department of Public and Occupational Health, Amsterdam Public Health, University of Amsterdam Medical Centers, Amsterdam, The Netherlands; c Department of Women’s and Children’s Health, Karolinska Institute, Stockholm, Sweden; d Institute for Global Health, University College London, London, UK; e West Africa Region Office, African Population Health Research Center, Dakar, Senegal; f Data Synergy and Evaluation Unit, African Population Health Research Center, Nairobi, Kenya; g Sciensano, Service of Lifestyle and Chronic Diseases, Brussels, Belgium; h Division of Endocrinology, Diabetes, and Metabolism, Department of Medicine, Johns Hopkins University School of Medicine, Baltimore, MD, USA

**Keywords:** sugar-sweetened beverages, type 2 diabetes, ultra-processed foods, non-communicable diseases, ecological analysis

## Abstract

**Background:**

Sugar-sweetened beverages (SSBs) are recognized contributors to the global rise in non-communicable diseases. While the link between SSB intake and adverse health outcomes is well established, long-term data from African countries are limited.

**Objective:**

To assess trends in SSB sales and their associations with type 2 diabetes (T2D) burden across nine African countries from 2010 to 2024.

**Methods:**

We conducted an ecological time-series analysis using national-level data from Cameroon, Côte d’Ivoire, Ethiopia, Ghana, Kenya, Morocco, Nigeria, South Africa, and Uganda. Annual changes in per capita and total SSB sales, national T2D prevalence, and the number of adults with T2D were analyzed. Country-specific multivariate Vector Autoregressive (MVAR) models estimated associations between SSB sales and T2D outcomes.

**Results:**

SSB sales rose across all countries, with the sharpest per capita increases in Cameroon (+173.8%), Nigeria (+119.1%), and Côte d’Ivoire (+88.5%). T2D trends varied: Ethiopia, Morocco, and South Africa showed rising prevalence and case numbers, while Ghana and Nigeria showed declines. Per capita SSB sales were significantly associated with adult T2D burden in Ghana (β = 0.41, *p* = 0.005) and Ethiopia (β = 0.37, *p* = 0.039. Total SSB volume was associated with T2D burden in Kenya (β = 0.49, *p* = 0.046) and with T2D prevalence in Nigeria, Morocco, and Côte d’Ivoire.

**Conclusions:**

Rising SSB sales may be contributing to the T2D burden in African countries. This calls for context-specific regulatory measures, such as fiscal taxes and front-of-pack labels.

## Paper Context

- **Main findings:** An ecological time-series analysis of nine African countries from 2010 to 2024 revealed that rising sugar-sweetened beverage sales are associated with an increasing burden of type 2 diabetes, particularly in countries undergoing rapid urbanization and dietary transitions.
- **Added knowledge:** This is the first multi-country time-series analysis in Africa to quantify temporal associations between national sugar-sweetened beverages sales and diabetes burden, using multivariate autoregressive modeling and harmonized regional data.
- **Global health impact for policy and action:** The findings support the use of regulatory measures such as sugar-sweetened beverages taxation, front-of-pack labeling, and marketing restrictions as viable tools to mitigate rising diabetes risk linked to food environment changes in low- and middle-income countries.

## Background

Non-communicable diseases (NCDs) are now the leading cause of death globally, accounting for more than 70% of all deaths, with over 80% of these occurring in low- and middle-income countries [1,2]. In Africa, this burden is growing rapidly due to urbanization, population aging, and changes in health behaviors [3–5]. Type 2 diabetes mellitus (T2D) is one of the fastest rising NCDs, with 24 million adults currently affected across the African continent, a number projected to reach 55 million by 2045 [6]. Considering Cameroon, Côte d’Ivoire, Ethiopia, Ghana, Kenya, Morocco, Nigeria, South Africa, and Uganda, the prevalence of diabetes ranges from 11.2% in South Africa to 3.7% in Uganda, with most countries reporting rates between 4% and 8% [6]. These figures reflect both the magnitude and heterogeneity of the diabetes burden across African regions [7,8].

This growing burden of NCDs is underpinned by the nutrition transition, shaped by industrialization, economic globalization, and urbanization, which has driven a shift from traditional, minimally processed diets toward energy-dense, ultra-processed foods (UPFs) high in added sugars, unhealthy fats, and refined carbohydrates, among other risk factors [9–11]. Research conducted in South Africa, Ghana, Uganda, and Kenya further highlights the widespread and aggressive marketing of unhealthy foods and beverages, particularly sugar-sweetened beverages (SSBs), across various food environment settings [12–16]. These conditions are especially pronounced in urban centers, where exposure to ultra-processed foods (UPFs) and SSBs is extensive and largely unregulated, contributing to rising obesity rates and an increased risk of diabetes [17,18].

Globally, robust evidence consistently demonstrates that regular consumption of SSBs and UPFs heightens the risk of obesity, insulin resistance, T2D, and other cardiometabolic conditions [19–22]. This trend aligns with findings in low- and middle-income countries (LMICs) experiencing increased rates of urbanization and industrialization [21]. Large-scale cohort studies in high-income countries (HICs) have reported up to a 26% increased diabetes risk among high SSB consumers, alongside elevated mortality and multimorbidity linked to UPF intake [23,24]. Furthermore, studies in African countries have shown similar associations, all connected to shifts in food environments, resulting in consequential cardiometabolic conditions, including obesity, T2D, and cardiovascular diseases [25–29]. Prior research has predominantly relied on cross-sectional data or focused on high-income settings, which limits its applicability to African contexts.

This study addresses a critical gap by presenting the first multi-country ecological time-series analysis of SSBs sales and diabetes burden across nine African countries. This offers region-specific longitudinal insights essential for informing effective and contextually relevant public health and nutrition policies. The study investigates the ecological association between SSBs consumption and diabetes burden in nine African countries from 2010 to 2024. It utilizes generalized least squares (GLS) models to assess within-country temporal trends and multivariate vector autoregressive (VAR) models to examine country-specific associations between SSBs consumption and diabetes indicators, including prevalence and adult case numbers.

## Methods

### Study design

This was an ecological time-series study based on annual country-level data from 2010 to 2024. The analysis was conducted to describe trends and explore associations between SSBs sales and T2D indicators across nine African countries.

### Study sites

**Figure 1.**
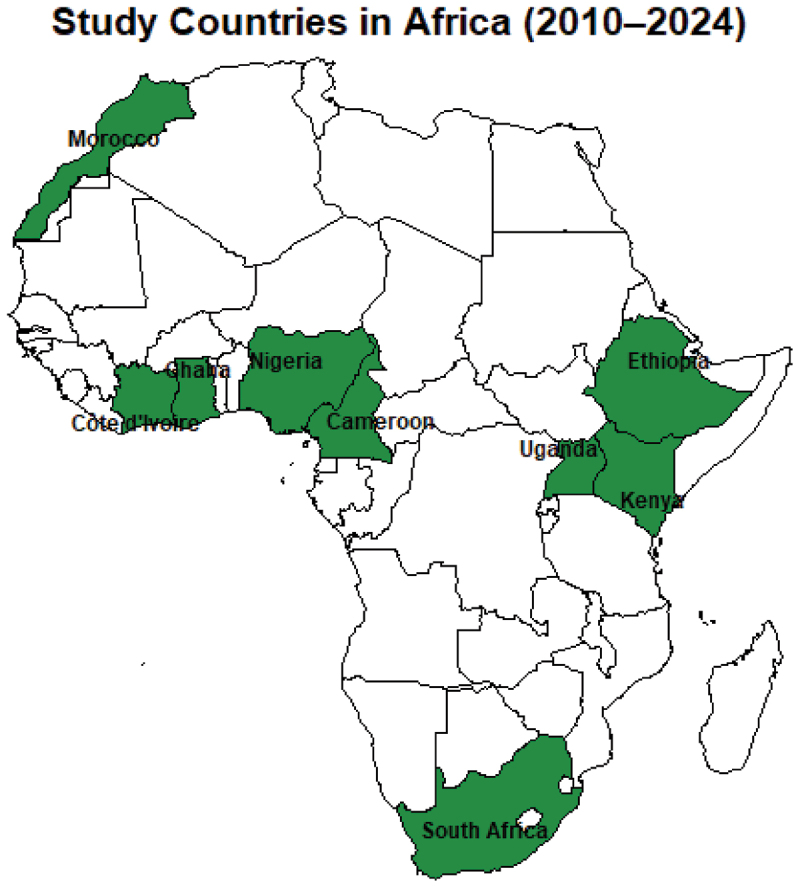
Map of study countries included in the ecological time-series analysis (2010–2024).

The study included data from nine purposively selected African countries: Cameroon, Côte d’Ivoire, Ethiopia, Ghana, Kenya, Morocco, Nigeria, South Africa, and Uganda (Figure 1). These countries were selected to ensure representation across the five African regions (West, East, Central, North, and Southern Africa) and based on the availability of continuous data of sales Volume data and per capita sales of interest over the 15-year period.

## Data sources and variables

### Diabetes indicators data

Data on national diabetes prevalence (%) and the number of adults with diabetes (aged 20–79 years, in thousands) were extracted from the International Diabetes Federation (IDF) Diabetes Atlas, using the 4th (2010) to 11th (2024) editions [30]. The IDF derives its estimates by synthesizing evidence from national health monitoring tools such as the WHO STEPS surveys and the peer-reviewed research literature [6]. The Atlas provides estimates that include both diagnosed and undiagnosed diabetes among adults aged 20–79 years, based on modelled data from population studies with biochemical measurements. This ensures that the prevalence values reflect the total diabetes burden rather than only the detected cases. The indicators in all the countries are similar and were derived using the same methodology. Only the country-level values presented in the regional summary tables for Africa were used. All extracted data reflect officially reported estimates for each year of publication. As the key Response variable, the T2D burden was assessed using two outcome indicators, extracted from official IDF Diabetes Atlas estimates. National diabetes prevalence (%) captures the proportion of adults aged 20–79 years diagnosed with diabetes each year, reflecting the relative burden of diabetes [6]. The number of adults with diabetes (in thousands) represented the absolute count of adults living with diabetes, reflecting the total population-level statistics.

### SSB sales volume data

SSBs sales data were obtained from Euromonitor International’s Passport Global Market Information Database [31]. Euromonitor International serves as a key source of standardized annual data on consumer goods, offering detailed information on sugar-sweetened beverage sales by volume and value, disaggregated by product category, brand, and distribution channel across countries, thereby facilitating robust analysis of market trends and consumption patterns [31]. SSBs are defined as non-alcoholic drinks containing added caloric sweeteners such as sucrose, high-fructose corn syrup, or fruit juice concentrates. This category includes carbonated soft drinks, fruit drinks (excluding 100% juice), energy drinks, sweetened teas, flavored waters, and other sugar-added beverages. The two measures extracted were total SSBs sales volume (million liters), representing national supply per year, and per capita SSBs consumption (liters/person/year), calculated by dividing total volume by the adult population to adjust for population size differences. Adult population denominators were obtained from the database, which compiles statistics from official national census bureaus, national and international demographic sources, and proprietary forecasting models that are updated annually [31]. These indicators reflect the availability and potential consumption of non-alcoholic SSBs such as carbonated drinks, sweetened juice products, and ready-to-drink teas. The analysis incorporated two primary exposure variables; Total SSBs sales (million liters) were defined as the annual national volume of SSBs sold, serving as a proxy for overall market-level supply and population-level exposure [31]. Per capita SSBs sales (liters/person/year) were derived by dividing the total volume by the adult population, providing a standardized measure that adjusts for country-specific population size.

### Urbanization and economic data

Country-level estimates of urbanization rates (expressed as the percentage of the total population living in urban areas) and GDP per capita (in current US dollars) were obtained from the World Bank’s World Development Indicators database [32]. These indicators were selected as key socioeconomic covariates due to their potential influence on both dietary behaviors and the epidemiology of T2D. Annual values from 2010 to 2024 were extracted for each of the nine countries for use in adjusted models to account for underlying macroeconomic and demographic trends [32]. To account for potential confounding, these variables were used `as covariates and included in all multivariable models. GDP per capita (current USD) was used as an economic indicator reflecting purchasing power and access to commodities, while urbanization rate (%) captured the proportion of the population residing in urban areas, a known modifier of dietary patterns and NCD risk. All variables were continuous and analyzed at the country-year level for the period 2010 to 2024.

## Data analysis

### Descriptive trends analysis of data from 2010 to 2024

To quantify longitudinal shifts in diabetes burden and SSBs sales, we calculated absolute and percentage changes between 2010 and 2024 for each country and indicator. Observed values for national diabetes prevalence (%), number of adults with diabetes (in thousands), per capita SSBs sales (liters/person/year), and total SSBs volume (million liters) were extracted for both endpoints. Absolute change was defined as the difference in values between 2024 and 2010, while percentage change was computed relative to 2010 levels.

Additionally, we conducted a country-specific time trend analysis using annual data from 2010 to 2024 across nine African countries. For each country and outcome variable, generalized least squares (GLS) regression models were employed, specifying year as the independent variable and incorporating an autoregressive (AR [1]) correlation structure to account for serial autocorrelation in the residuals. This approach ensures more reliable estimation of standard errors by adjusting for the temporal dependence inherent in time-series data. For each fitted model, we derived the intercept (reflecting the baseline estimate in 2010), the slope (indicating the annual rate of change), and the corresponding p-value for statistical significance. The indicators assessed included national T2D prevalence (%), number of adults with T2D (in thousands), per capita and total SSBs sales, GDP per capita, and urbanization rate. Analyses were performed separately for each country to capture distinct national trajectories over the study period.

### Multivariate Vector Autoregressive (MVAR) time-series analysis

To examine the temporal association between SSBs sales and T2D outcomes within each country, we employed multivariate Vector Autoregressive (VAR) modelling. VAR models are well-suited for evaluating interdependence among time-series variables, where each variable is treated as endogenous and influenced by its past values and those of other variables in the system. This framework allows for flexible estimation of lagged relationships without imposing strict causality assumptions. For each of the nine countries, we estimated separate multivariate VAR models using annual data from 2010 to 2024. Two T2D-related outcomes were modelled independently: national T2D prevalence (%) and the number of adults with T2D (in thousands). In each specification, the key predictors were per capita SSBs sales (liters/person/year) and total SSBs sales volume (million liters). Each VAR model included all three variables and was estimated with a fixed intercept and no deterministic trend.

Prior to model fitting, the time series for each country was log-transformed and tested for stationarity using the Augmented Dickey-Fuller (ADF) and Kwiatkowski-Phillips-Schmidt-Shin (KPSS) tests. Variables were retained at levels if stationarity was supported or if differencing risked loss of long-term interpretability. The optimal lag order for each country-specific model was selected using the Akaike Information Criterion (AIC), with a maximum lag of 3 years. In addition, Model diagnostics were also conducted to confirm the adequacy of the VAR specifications. We verified model stability by checking that all eigenvalues of the companion matrix lay within the unit circle and assessed residual autocorrelation using Portmanteau and Breusch – Godfrey LM tests. This was to ensure that the models were dynamically stable, with shocks dissipating over time, and that residuals were free from serial dependence, confirming the appropriateness of the selected lag length.

The data were analyzed using R Software Version 4.5.0, and the Statistical significance level was set at α = 0.05. Model estimation was conducted using the vars package in R. Coefficients, 95% confidence intervals, and p-values were extracted and reported for all predictors.

## Results

### Changes in SSB sales and diabetes indicators (2010–2024)

Table 1 presents observed values in 2010 and 2024, along with absolute and percentage changes, for T2D indicators and SSBs sales. It highlights inter-country variation in the magnitude of change over the study period.

**Table 1.** Observed values and changes in SSBs sales and T2D indicators between 2010 and 2024 across nine African countries.

Variable	Country	2010	2024	Absolute Change	Percentage Change (%)
National T2D Prevalence (%)	Cameroon	4.40	5.60	1.20	27.27
	Cote d’ Ivoire	4.00	3.70	−0.30	−7.50
	Ethiopia	2.00	3.60	1.60	80.00
	Ghana	3.60	1.70	−1.90	−52.78
	Kenya	2.80	2.80	0.00	0.00
	Morocco	7.60	11.60	4.00	52.63
	Nigeria	3.90	2.80	−1.10	−28.21
	South Africa	4.50	6.10	1.60	35.56
	Uganda	1.70	1.70	0.00	0.00
Adults with T2D (000s)	Cameroon	415.30	774.20	358.90	86.42
	Cote d’ Ivoire	393.90	529.20	135.30	34.35
	Ethiopia	826.00	2297.30	1471.30	178.12
	Ghana	458.30	317.40	−140.90	−30.74
	Kenya	519.10	813.30	294.20	56.68
	Morocco	1512.80	2881.00	1368.20	90.44
	Nigeria	2819.10	2988.50	169.40	6.01
	South Africa	1283.40	2324.20	1040.80	81.10
	Uganda	224.10	369.10	145.00	64.70
Per Capita SSBs Sales (Litres)	Cameroon	16.80	46.00	29.20	173.81
	Cote d’ Ivoire	6.10	11.50	5.40	88.52
	Ethiopia	11.80	14.90	3.10	26.27
	Ghana	192.70	302.20	109.50	56.82
	Kenya	15.90	23.30	7.40	46.54
	Morocco	38.00	46.80	8.80	23.16
	Nigeria	104.20	228.30	124.10	119.10
	South Africa	89.10	128.80	39.70	44.56
	Uganda	30.10	36.50	6.40	21.26
Total SSBs Sales (Million Litres)	Cameroon	330.30	1340.00	1009.70	305.69
	Cote d’ Ivoire	137.80	368.20	230.40	167.20
	Ethiopia	1072.60	1970.50	897.90	83.71
	Ghana	4908.10	10,403.20	5495.10	111.96
	Kenya	660.20	1313.60	653.40	98.97
	Morocco	1233.50	1781.00	547.50	44.39
	Nigeria	17,357.90	53,125.20	35,767.30	206.06
	South Africa	4664.10	8241.70	3577.60	76.71
	Uganda	976.30	1828.00	851.70	87.24

Between 2010 and 2024, all nine countries experienced a notable increase in per capita SSBs sales. The sharpest relative growth was observed in Cameroon (+173.8%), Nigeria (+119.1%), and Côte d’Ivoire (+88.5%). In absolute terms, Nigeria showed the greatest increase of 124.1 liters per capita. In terms of total SSBs sales volume, all countries saw significant increases, driven by both per capita sales and population growth. Nigeria led with an increase of 35.77 billion liters (+206.1%), followed by Ghana (+5.50 billion liters, +112.0%) and Cameroon (+1.01 billion liters, +305.7%).

Changes in T2D prevalence had more variability with Morocco (+4.00%, +52.6%), Ethiopia (+1.60%, +80.0%), and South Africa (+1.60%, +35.6%) recording the largest increases. In contrast, Ghana (−1.90%, −52.8%) and Nigeria (−1.10%, −28.2%) showed declines in prevalence. Notably, countries like Kenya and Uganda maintained a stable prevalence of T2D.

Regarding the absolute number of adults with T2D, all countries except Ghana reported increases. The most significant absolute increases were recorded in Ethiopia (+1.47 million, +178.1%), Morocco (+1.37 million, +90.4%), and South Africa (+1.04 million, +81.1%).

### Descriptive trends in T2D, SSBs sales, economic growth, and urbanization (2010–2024)

Table 2 summarizes the estimated annual changes in T2D burden, SSBs sales, gross domestic product (GDP) per capita, and urbanization rates between 2010 and 2024 across nine African countries using country-specific linear trend models adjusted for autocorrelation.

**Table 2.** Estimated annual changes in T2D, SSBs sales, GDP, and urbanization from linear trend models (2010–2024) across nine African countries.

Varia	Country	Intercept (2010)	Slope (Annual Change)	*P*-value
Adults with T2D (000’s)	Cameroon	−42240.09	21.24	< 0.001
	Ghana	25910.04	−12.66	0.120
	Morocco	−194923.73	97.73	0.122
	South Africa	−197820.26	99.34	0.477
	Ethiopia	−157205.64	78.83	0.056
	Uganda	−19293.03	9.77	0.587
	Nigeria	−24828.57	13.72	0.896
	Kenya	−22547.56	11.52	0.283
	Cote d’ Ivoire	−19031.35	9.66	0.762
GDP Per Capita (USD)	Cameroon	−37070.42	19.16	0.010
	Ghana	−127386.4	64.15	0.037
	Morocco	−89931.62	46.3	0.003
	South Africa	277694.39	−134.25	0.089
	Ethiopia	−134827.54	67.24	0.002
	Uganda	−24849.96	12.77	0.326
	Nigeria	113471.65	−55.19	0.158
	Kenya	−122491.87	61.48	0.061
	Cote d’ Ivoire	−145045.35	72.92	< 0.001
National T2D Prevalence (%)	Cameroon	−87.33	0.05	0.164
	Ghana	322.07	−0.16	0.021
	Morocco	−601.49	0.3	0.001
	South Africa	−356.36	0.18	0.608
	Ethiopia	−88.15	0.05	0.579
	Uganda	38.59	−0.02	0.866
	Nigeria	162.56	−0.08	0.511
	Kenya	103.12	−0.05	0.265
	Cote d’ Ivoire	77.26	−0.04	0.867
Per Capita SSB Sales (Liters)	Cameroon	−4175.49	2.09	0.007
	Ghana	−15528.37	7.82	0.005
	Morocco	−1225.43	0.63	0.113
	South Africa	−5610.69	2.84	0.009
	Ethiopia	−433.27	0.22	0.462
	Uganda	−826.15	0.43	0.006
	Nigeria	−17713.01	8.86	< 0.001
	Kenya	−1046.53	0.53	0.001
	Cote d’ Ivoire	−769.19	0.39	0.039
Total SSB Sales (Million liters)	Cameroon	−144633.93	72.12	< 0.001
	Ghana	−784031.3	392.51	< 0.001
	Morocco	−77371.86	39.11	0.018
	South Africa	−509630.56	255.88	< 0.001
	Ethiopia	−128033.13	64.24	0.080
	Uganda	−121303.5	60.84	0.009
	Nigeria	−5117804.17	2554.81	< 0.001
	Kenya	−93149.37	46.67	< 0.001
	Cote d’ Ivoire	−33683.32	16.83	< 0.001
Urbanization Rate (%)	Cameroon	−1062.55	0.55	< 0.001
	Ghana	−1173.95	0.61	< 0.001
	Morocco	−961.34	0.51	< 0.001
	South Africa	−885.35	0.47	< 0.001
	Ethiopia	−821.14	0.42	< 0.001
	Uganda	−1041.61	0.53	< 0.001
	Nigeria	−1507.09	0.77	< 0.001
	Kenya	−831.4	0.43	< 0.001
	Cote d’ Ivoire	−788.26	0.42	< 0.001

Cameroon (β = 21.24, *p* < 0.001) was the only country with a statistically significant increase in the number of adults with T2D. While Ethiopia (β = 78.83, *p* = 0.056) and Morocco (β = 97.73, *p* = 0.122) showed large positive slopes, these did not reach statistical significance. For T2D prevalence, Ghana showed a significant decline (β = −0.16, *p* = 0.021), whereas Morocco experienced a significant increase (β = 0.30, *p* = 0.001).

Per capita SSBs sales increased significantly in all countries except Ethiopia and Morocco. The most pronounced increases were in Nigeria (β = 8.86, *p* < 0.001), Ghana (β = 7.82, *p* = 0.005), and Kenya (β = 0.53, *p* = 0.001). Similarly, total SSBs sales volumes rose significantly across all countries, with the largest annual increases in Nigeria (β = 2554.81, *p* < 0.001), Ghana (β = 392.51, *p* < 0.001), and South Africa (β = 255.88, *p* < 0.001).

For GDP per capita, significant upward trends were observed in Côte d’Ivoire (β = 72.92, *p* < 0.001), Ethiopia (β = 67.24, *p* = 0.002), Morocco (β = 46.30, *p* = 0.003), Ghana (β = 64.15, *p* = 0.037), and Cameroon (β = 19.16, *p* = 0.010). Urbanization rates increased significantly in all nine countries (*p* < 0.001), with the steepest trends in Nigeria (β = 0.77) and Ghana (β = 0.61), reflecting rapid urban transformation across the region.

### Multivariate country-level Vector Autoregressive (MVAR) models

**Figure 2.**
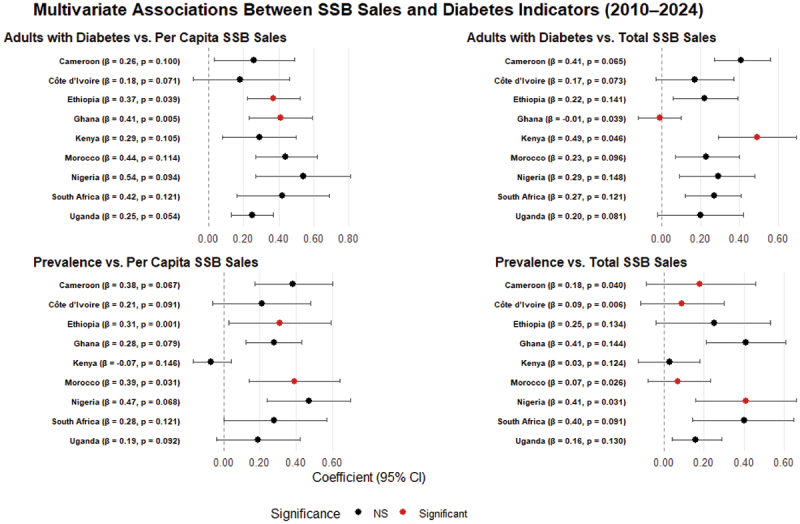
Forest plot on the associations between SSB sales and diabetes indicators.

Figure 2 presents a forest plot on country-specific associations between SSBs sales (both per capita and total volume) and T2D outcomes, including national prevalence and the adult diabetic population, based on separate multivariate VAR models for each country.

The full regression outputs are presented in Supplementary Table S1. Associations between total SSBs volume and national T2D prevalence were variable in the 9 African countries (Figure 2). Significant or strong positive relationships were observed in Nigeria (β = 0.41, 95% CI: 0.16 to 0.66, *p* = 0.031) and Morocco (β = 0.07, *p* = 0.026) (Figure 2). Ghana also had a relatively large coefficient (β = 0.41), though the association was marginal (*p* = 0.144). Smaller or borderline effects were reported in Cameroon (β = 0.18, *p* = 0.040) and Côte d’Ivoire (β = 0.09, *p* = 0.006), while Kenya showed no meaningful association (β = 0.03, *p* = 0.124) (Figure 2). These findings suggest country-level variation in the relevance of national SSBs volumes to T2D prevalence.

Significant associations were observed between per capita SSBs sales and the number of adults with T2D in Ghana (β = 0.41, 95% CI: 0.23 to 0.59, *p* = 0.005) and Ethiopia (β = 0.37, 95% CI: 0.22 to 0.52, *p* = 0.039). Notably large coefficients were also observed in Nigeria (β = 0.54, 95% CI: 0.27 to 0.81, *p* = 0.094) and Morocco (β = 0.44, *p* = 0.114) (Figure 2). Borderline or associations were seen in Kenya (β = 0.29, *p* = 0.105), Uganda (*p* = 0.054), and Cameroon (*p* = 0.100) (Figure 2).

The relationship between total SSBs sales volume and adult T2D burden was strongest in Kenya (β = 0.49, 95% CI: 0.29 to 0.69, *p* = 0.046), with consistent borderline non-significant positive associations also observed in South Africa, Morocco, and Ethiopia (Figure 2). While these associations were not always statistically significant, the direction and magnitude suggest a potential influence of total SSB sales on T2D burden. In contrast, Ghana showed a negative significant association (β = −0.01, *p* = 0.039), indicating heterogeneity in how population-level SSB exposure may relate to diabetes outcomes across countries.

## Discussion

The study aimed to investigate the trends and the ecological association between SSBs sales, an indirect measure of consumption, and the burden of T2D across nine African countries from 2010 to 2024. We observed statistically significant increases in both per capita and total SSBs consumption across all countries, with Nigeria, Ghana, and Cameroon experiencing particularly rapid surges. Trends in T2D indicators, however, were heterogeneous. Ethiopia, Morocco, and South Africa recorded substantial increases in both the prevalence and total number of adults with T2D, while Ghana and Nigeria reported declines in prevalence, suggesting that SSBs availability has grown more consistently compared to the T2D burden in those countries. Furthermore, we found significant associations between SSBs consumption and T2D outcomes in several countries. These associations were stronger and more consistent for the absolute number of adults with T2D than for national prevalence rates.

Our findings are consistent with global trends observed over the last decade, showing increased awareness of the growing sales and consumption of SSBs and other UPFs in LMICs. Previous studies highlight the rapid increase in SSBs penetration in LMICs, with multinational beverage companies expanding into African markets [33,34]. Another study underscored how ultra-processed products, including SSBs, have quickly gained popularity in regions undergoing nutrition transition, such as African economies [4,34]. Country-specific studies have consistently shown that increased industrialization and the changing food environments are major contributors to individual SSBs consumption. This trend is largely driven by the increasing affordability and accessibility of unhealthy foods [11,34,35]. For instance, a study in three SSA countries, which assessed SSBs consumption among women and dietary transitions, revealed significantly elevated intake levels of high-energy-dense food consumption in more urbanized areas [5,36]. Additionally, the Kenya Demographic Health Survey 2022 indicates that 70.4% of women and 49% of children aged 6–23 months consumed SSBs [37]. These patterns collectively highlight a broader shift in dietary behaviors across LMICs. Here, structural and commercial forces have combined to normalize SSBs consumption, particularly among vulnerable groups such as women and young children.

We observed an increase in the burden of T2D in most countries, consistent with global analyses indicating a substantial rise in T2D prevalence, doubling from 6.8% in 1990 to 14.1% in 2022, with case numbers increasing from approximately 30 million in 2010 to 54 million [38,39]. In African countries, T2D prevalence has risen from 6.4% in 1990 to 10.5% in 2022 [40,41]. Country-level studies in Nigeria, Ethiopia, Morocco, and Ghana show similar increasing patterns across Africa, though Nigeria’s prevalence has a downward trend despite high crude numbers, and Ghana experienced a negative change, possibly reflecting the impact of its SSBs tax [1,6]. Country-level studies in Nigeria, Ethiopia, Morocco, and Ghana show similar increasing patterns across Africa [42–44]. Nigeria’s prevalence has a downward trend despite high crude numbers, and Ghana experienced a negative change, possibly reflecting the impact of its SSB tax [39,45,46]. In addition to the potential effects of recent health system initiatives to improve diabetes awareness, screening, and case reporting, demographic and lifestyle changes may also play a role. Furthermore, shifts in the modelling inputs and methods between successive IDF Atlas editions could partly account for the lower prevalence estimates, underscoring the need for careful interpretation.

Our time-series multivariate analysis found significant associations between SSBs consumption and Type 2 Diabetes [T2D] indicators in some countries. This aligns with other research, showing that SSBs contribute to 9.8% of T2D and 3.1% of cardiovascular disease cases [47]. High-income country studies also link increased T2D and cardiovascular diseases to UPFs, including SSBs [23,24]. SSA countries bear a high burden, with 21.5% of T2D and 10.5% of cardiovascular disease cases attributed to SSBs [47]. African studies also show a link between SSBs affordability, higher consumption, and increased overweight and obesity rates, a key T2D risk factor [27,35]. Further studies in Sudan, Nigeria, South Africa, and Kenya reinforce the association between UPF consumption and cardiometabolic conditions like obesity and T2D [26,28,29]. These findings underscore the growing global and local evidence linking SSBs consumption to rising T2D among other cardiometabolic risks. This highlights the urgent need for targeted policy action in African settings experiencing rapid nutrition transition. Addressing the health impacts of SSBs is crucial for curbing the long-term NCD burden as the region faces increased exposure to UPFs and beverages.

### Policy implications

These findings present several critical policy implications in African countries. The continued rise in SSBs consumption across all countries, despite varying trends in T2D prevalence, underscores the need for proactive, multisectoral interventions. These interventions are crucial to prevent further increases in diet-related NCDs. Countries showing emerging or significant associations between SSBs consumption and T2D outcomes, such as Ethiopia, Ghana, Morocco, and Nigeria, are in a strong position to implement preventive policy measures. These measures could include fiscal interventions like excise taxes on SSBs, which have been shown to reduce purchase and consumption in other settings. Evaluations of SSBs taxes in Mexico, South Africa, and the United Kingdom show that such policies can significantly lower intake while encouraging industry reformulation towards lower sugar content [25,48–51]. Additionally, front-of-package (FOP) nutrition labeling can improve consumer awareness of sugar content and encourage healthier purchasing decisions. Interpretive labeling schemes, such as warning labels, have been linked to better consumer understanding and small shifts in food purchases away from high-sugar products. Implementing these schemes in African markets would support taxation policies and serve as an important tool for public information [49]. Implementing such schemes in African markets would complement taxation policies and serve as a critical tool for informing the public. Moreover, regulating the marketing of SSBs, especially to children and adolescents, is essential. The proliferation of aggressive marketing practices in urbanizing African settings has contributed to the normalization of SSBs consumption among youth [12,14,15,52]. Restricting SSBs advertising in school zones, on television during peak hours, and online platforms is consistent with WHO recommendations and could help reduce exposure to unhealthy product promotions [53,54]. Such policies are urgently needed in rapidly urbanizing and economically transitioning African countries, where shifts in food environments are accelerating dietary transitions and increasing vulnerability to NCDs in children and adults.

### Strengths, limitations, and future research

This study has several strengths. It leverages 15 years of harmonized, country-level data across nine countries, incorporates both absolute and relative T2D measures, and applies robust multivariate time-series models. The use of GLS and MVAR modeling allowed for the correction of autocorrelation and provided more reliable effect estimates. A key limitation of the IDF Diabetes Atlas data is the reliance on extrapolated estimates for countries lacking recent or nationally representative data. In such cases, prevalence figures are derived from countries with similar economic, demographic, or linguistic profiles, which may introduce uncertainty or bias and limit the precision of country-specific estimates [6]. The second limitation includes the ecological design, which limits causal inference at the individual level, potential data quality issues in SSBs sales estimates, and the inability to control for all the confounders, such as physical activity or healthcare access. Moreover, attempts to adjust the models for GDP per capita and urbanization led to poor model stability. Most adjusted models failed to converge or yielded non-estimable coefficients with wide confidence intervals and missing p-values. This instability is largely attributable to high collinearity between urbanization, GDP, and SSBs sales, which limits the ability to isolate independent effects in time-series models, especially when working with short panels (2010–2024). Furthermore, while Euromonitor and IDF represent the most comprehensive and comparable data sources available, SSB sales data may underestimate true consumption by excluding informal and home-prepared beverages, and IDF estimates rely on modeled data that may vary in quality across countries. These factors could affect the precision of our estimates. While our analysis provides novel evidence, it was not possible to incorporate formal sensitivity analyses across countries because of the short observation period and the absence of harmonized contextual variables, such as obesity prevalence, dietary patterns, or fiscal policies like SSB taxation. Future work with extended time series and richer, standardized policy and lifestyle data will be crucial to examine the robustness of these associations and to better understand how fiscal and regulatory measures may modify the relationship between food environments and diabetes burden in African settings. Future studies incorporating individual-level data and longer timeframes are warranted to further unpack these relationships and inform context-appropriate public health responses

## Conclusions

This study underscores a concerning rise in SSBs sales, implying increased consumption across African countries, occurring alongside divergent trends in T2D prevalence and burden. While T2D outcomes have not uniformly tracked the surge in SSBs availability, emerging positive associations in several countries raise public health concerns. The evidence supports the need for urgent preventive action, particularly in countries experiencing rapid urbanization and dietary transitions. To mitigate the growing burden of nutrition-related NCDs, governments should consider implementing comprehensive regulatory measures such as SSBs taxation, front-of-pack labeling, marketing restrictions, and public education campaigns to curb consumption and reduce health risks. These findings reinforce calls for proactive policy responses tailored to the evolving food environments in the African context.

## Supplementary Material

Supplementary Tables_GHAp1.docx

## Data Availability

The datasets used and/or analysed during the current study are available from the corresponding author on reasonable request. Additionally, the data is available online on the International Diabetes Federation website (https://idf.org/) and Euromonitor International website (https://www.euromonitor.com/).
