# Supplementary material for: Trends in sales of sugar-sweetened beverages and associated type 2 diabetes burden in nine African countries: an ecological time-series analysis
Source: Glob Health Action. 2025 Oct 9;18(1):2568283. doi: 10.1080/16549716.2025.2568283 (PMC12512766; doi:10.1080/16549716.2025.2568283)
Supplement: Supplementary Tables_GHAp1.docx [file ZGHA_A_2568283_SM2389.docx]

**Supplementary Tables**

**Supplementary Table 1: Multivariate associations between sugar-sweetened beverage (SSB) sales and diabetes indicators by country (2010–2024)**

| **Model** | **Country** | **Coefficient (95% CI)** | **P-value** |
| --- | --- | --- | --- |
| **Prevalence vs. Per Capita SSB Consumption** | Cameroon | 0.38 (0.17, 0.60) | 0.067 |
|  | Cote d’ Ivoire | 0.21 (-0.06, 0.48) | 0.091 |
|  | Ethiopia | 0.31 (0.03, 0.59) | 0.001 |
|  | Ghana | 0.28 (0.12, 0.43) | 0.079 |
|  | Kenya | -0.07 (-0.17, 0.04) | 0.146 |
|  | Morocco | 0.39 (0.14, 0.64) | 0.031 |
|  | Nigeria | 0.47 (0.24, 0.70) | 0.068 |
|  | South Africa | 0.28 (-0.00, 0.57) | 0.121 |
|  | Uganda | 0.19 (-0.04, 0.42) | 0.092 |
| **Prevalence vs. Total SSB Volume** | Cameroon | 0.18 (-0.09, 0.46) | 0.040 |
|  | Cote d’ Ivoire | 0.09 (-0.12, 0.30) | 0.006 |
|  | Ethiopia | 0.25 (-0.04, 0.53) | 0.134 |
|  | Ghana | 0.41 (0.21, 0.61) | 0.144 |
|  | Kenya | 0.03 (-0.13, 0.18) | 0.124 |
|  | Morocco | 0.07 (-0.08, 0.23) | 0.026 |
|  | Nigeria | 0.41 (0.16, 0.66) | 0.031 |
|  | South Africa | 0.40 (0.14, 0.65) | 0.091 |
|  | Uganda | 0.16 (0.04, 0.29) | 0.130 |
| **Adults with Diabetes vs. Per Capita SSB Consumption** | Cameroon | 0.26 (0.03, 0.49) | 0.100 |
|  | Cote d’ Ivoire | 0.18 (-0.09, 0.46) | 0.071 |
|  | Ethiopia | 0.37 (0.22, 0.52) | 0.039 |
|  | Ghana | 0.41 (0.23, 0.59) | 0.005 |
|  | Kenya | 0.29 (0.08, 0.50) | 0.105 |
|  | Morocco | 0.44 (0.27, 0.62) | 0.114 |
|  | Nigeria | 0.54 (0.27, 0.81) | 0.094 |
|  | South Africa | 0.42 (0.16, 0.69) | 0.121 |
|  | Uganda | 0.25 (0.13, 0.37) | 0.054 |
| **Adults with Diabetes vs. Total SSB Volume** | Cameroon | 0.41 (0.27, 0.56) | 0.065 |
|  | Cote d’ Ivoire | 0.17 (-0.03, 0.37) | 0.073 |
|  | Ethiopia | 0.22 (0.06, 0.39) | 0.141 |
|  | Ghana | -0.01 (-0.12, 0.10) | 0.039 |
|  | Kenya | 0.49 (0.29, 0.69) | 0.046 |
|  | Morocco | 0.23 (0.07, 0.40) | 0.096 |
|  | Nigeria | 0.29 (0.09, 0.48) | 0.148 |
|  | South Africa | 0.27 (0.12, 0.41) | 0.121 |
|  | Uganda | 0.20 (-0.02, 0.42) | 0.081 |
